# Effect of Chitosan Phonophoresis on Cervical Pain, Disability and Cervical Range of Motion in Smartphone-Addicted Users Aged 16–19 Years: Randomized Controlled Trial

**DOI:** 10.3390/healthcare14213626

**Published:** 2026-10-26

**Authors:** Samar Salem Korney Ramdan, Lama Saad El-Din Mahmoud, Sara Abd El-wahab Fetoh, Shahesta Ahmed Osama, Mohamed A. Abdel Ghafar, Safaa M. Elkholi, Baher A. Daihom, Hala N. ElShagea, Ahmed Sayed Mahmoud Tolba

**Affiliations:** 1 Department of Pediatrics, Faculty of Physical Therapy, October 6 University, Giza 12585, Egypt; samar.salem.pt@o6u.edu.eg (S.S.K.R.); ahmed.sayed.pt@o6u.edu.eg (A.S.M.T.); 2 Department of Physical Therapy for Neurology and Neurosurgery, Faculty of Physical Therapy, October 6 University, Giza 12585, Egypt; lama.elsedawy.pt@o6u.edu.eg; 3 Department of Basic Science, Faculty of Physical Therapy, October 6 University, Giza 12585, Egypt; sara.abdelwaha.pt@o6u.edu.eg; 4 Department of Physiotherapy, Faculty of Allied Medical Sciences, Al-Hussein Bin Talal University, Ma’an 71111, Jordan; 5 Department of Orthopedics, Faculty of Physical Therapy, October 6 University, Giza 12585, Egypt; shahesta.ahmed.pt@o6u.edu.eg; 6 Physical Therapy Program, Batterjee Medical College, Jeddah 21442, Saudi Arabia; 7 Department of Rehabilitation Sciences, College of Health and Rehabilitation Sciences, Princess Nourah bint Abdulrahman University, P.O. Box 84428, Riyadh 11671, Saudi Arabia; 8 Department of Pharmaceutics and Industrial Pharmacy, Faculty of Pharmacy, Cairo University, Cairo 11562, Egypt; baher.daihom@pharma.cu.edu.eg; 9 Department of Pharmaceutics and Industrial Pharmacy, Faculty of Pharmacy, Ahram Canadian University, Giza 12451, Egypt; hala.nehad@acu.edu.eg; 10 Department of Physical Therapy, Faculty of Allied Medical Sciences, Zarqa University, Zarqa 13132, Jordan

**Keywords:** cervical pain, smartphone addiction, chitosan, phonophoresis, neck disability, cervical range of motion

## Abstract

**Background and Purpose:** Prolonged smartphone use may contribute to cervical pain through sustained muscular overload and increased nociceptive input. This study investigated the effects of adding chitosan phonophoresis to conventional physical therapy on pain, disability, and cervical range of motion (ROM) in smartphone-addicted adolescents with cervical pain. **Methods:** Thirty-two participants aged 16–19 years with smartphone addiction and cervical pain were randomly assigned to a chitosan phonophoresis group or a control group. Outcomes were assessed before and after treatment using the Neck Disability Index (NDI), Visual Analog Scale for Pain (VAS-P), and cervical range-of-motion (CROM) device. **Results:** Both groups improved significantly after treatment. Compared with the control group, the chitosan phonophoresis group showed greater reductions in VAS-P (MD = −1.00, 95% CI: −1.59 to −0.41; *p* = 0.002) and NDI (MD = −3.13, 95% CI: −5.00 to −1.23; *p* = 0.002). Greater improvements were also observed in cervical ROM, including flexion (MD = 5.63°, 95% CI: 2.58 to 8.67), extension (MD = 6.25°, 95% CI: 1.90 to 10.59), right bending (MD = 3.75°, 95% CI: 1.74 to 5.76), left bending (MD = 4.38°, 95% CI: 1.95 to 6.79), right rotation (MD = 6.25°, 95% CI: 3.70 to 8.80), and left rotation (MD = 6.25°, 95% CI: 3.19 to 9.30; all *p* < 0.001). **Conclusions:** Adding chitosan phonophoresis to conventional physical therapy produced greater improvements in cervical pain, disability, and ROM. However, between-group differences in pain and disability were below commonly reported thresholds for clinically important change. Chitosan phonophoresis may be a useful adjunct in managing cervical disorders associated with prolonged smartphone use.

## 1. Introduction

Excessive smartphone use has become a widespread behavioral and musculoskeletal health concern, particularly among adolescents and young adults, who are highly exposed to prolonged daily screen time and have elevated rates of smartphone dependence [1]. Sustained smartphone use commonly places the cervical spine in a flexed, forward-head posture, increasing compressive and repetitive stress on cervical structures and contributing to what is often termed text neck syndrome [2]. Neck pain is among the most frequently reported musculoskeletal complaints among smartphone users, often accompanied by disability, reduced cervical mobility, postural deviations, and impaired muscle performance [3]. Prolonged cervical flexion and faulty posture appear to alter cervical alignment, irritate surrounding soft tissues, and promote muscular imbalance, reduced endurance, and proprioceptive dysfunction, which may collectively aggravate pain and functional limitations [4].

Recent trials indicate that conservative interventions, such as corrective exercises, deep-cervical-flexor training, stabilization programs, proprioceptive training, and ergonomic modifications, can reduce pain and disability in smartphone-related neck disorders [4,5,6]. Ergonomic and app-based strategies also appear to support posture correction, adherence, and self-management, with improvements reported in forward head posture, endurance, and disability outcomes [7,8]. Conventional physical therapy for neck pain commonly uses a multimodal approach targeting pain, muscle tightness, flexibility, and cervical postural and motor control. Therapeutic exercise and manual therapy have demonstrated beneficial effects on pain, range of motion (ROM), and disability in individuals with neck pain [9,10,11].

Ultrasound is a widely used physical therapy modality, and phonophoresis extends its application by enhancing the transdermal delivery of topical agents into deeper tissues [12,13]. In cervical and related neuromusculoskeletal conditions, phonophoresis has been associated with reductions in pain and disability, and when combined with exercise or manual therapy, it may produce greater functional improvements than exercise alone [13,14]. However, evidence is not uniformly favorable across formulations, as one randomized trial on myofascial pain syndrome found that phonophoresis improved symptoms but was not superior to therapeutic ultrasound alone [12].

Chitosan, a naturally occurring polysaccharide, is non-toxic, biocompatible, and biodegradable and has several potential medical applications. It has been proposed to reduce inflammatory activity by suppressing pro-inflammatory cytokines [15]. Chitosan is produced through the partial deacetylation of chitin, resulting in a copolymer containing N-acetylglucosamine and glucosamine units. Its properties, including stability at reduced pH, mucoadhesive capacity, and the ability to facilitate absorption across tight junctions, enable its use in the encapsulation and delivery of therapeutic proteins and peptides across mucosal barriers [16].

Chitosan has attracted interest because of its proposed neuroregenerative and tissue-repair properties, and recent randomized evidence suggests that chitosan phonophoresis, when used as an adjunct to standard hand therapy, can improve nerve conduction, pain, and functional outcomes in peripheral nerve entrapment disorders [17]. In addition, previous clinical research has suggested potential therapeutic applications of chitosan in musculoskeletal and pain-related conditions. Clinical studies of medical and carboxymethyl chitosan have reported improvements in pain and functional outcomes in patients with knee osteoarthritis [18].

Given the high prevalence of cervical pain and disability among smartphone-addicted users, along with the postural and neuromuscular alterations associated with prolonged smartphone use, there is a need for effective, non-invasive therapeutic interventions. Although conventional multimodal physical therapy can address several of the impairments associated with cervical pain, the potential additional benefit of incorporating chitosan phonophoresis into such a rehabilitation program has not been adequately investigated in smartphone-addicted users. Therefore, this randomized controlled trial aimed to evaluate the clinical effects of chitosan phonophoresis, when added to conventional physical therapy, on cervical pain and disability in smartphone-addicted users. The findings may provide evidence useful for designing an innovative, non-invasive rehabilitation strategy for improving pain management and functional outcomes.

## 2. Materials and Methods

### 2.1. Study Design

This study was designed as a two-arm, parallel-group randomized controlled trial with concealed allocation and blinded assessment of cervical range of motion (CROM). Pain intensity and neck disability were assessed using participant-reported questionnaires (VAS-P and NDI), and participants were aware of their treatment allocation. The trial was reported in accordance with the CONSORT guidelines (Table S1). The trial was reported in accordance with the CONSORT guidelines (Table S1). Participants were randomly allocated to either the control group or the chitosan phonophoresis group and underwent their assigned interventions over a four-week treatment period.

### 2.2. Randomization

The study used stratified randomization to allocate participants to either the Chitosan phonophoresis group or control group. Stratification was performed according to gender to ensure a balanced distribution of participants between the groups. Within each stratum, participants were randomly allocated using permuted blocks of four based on a computer-generated random sequence. To ensure allocation concealment, group assignments were placed in sequentially numbered, sealed, opaque envelopes. The envelopes were opened only after confirmation of participant eligibility and completion of the baseline assessment. An independent third party who was not involved in participant recruitment, outcome assessment, or intervention administration, opened the envelopes and assigned participants to their respective groups. This procedure was used to minimize the risk of selection bias and maintain the integrity of the randomization process.

**Figure 1:**
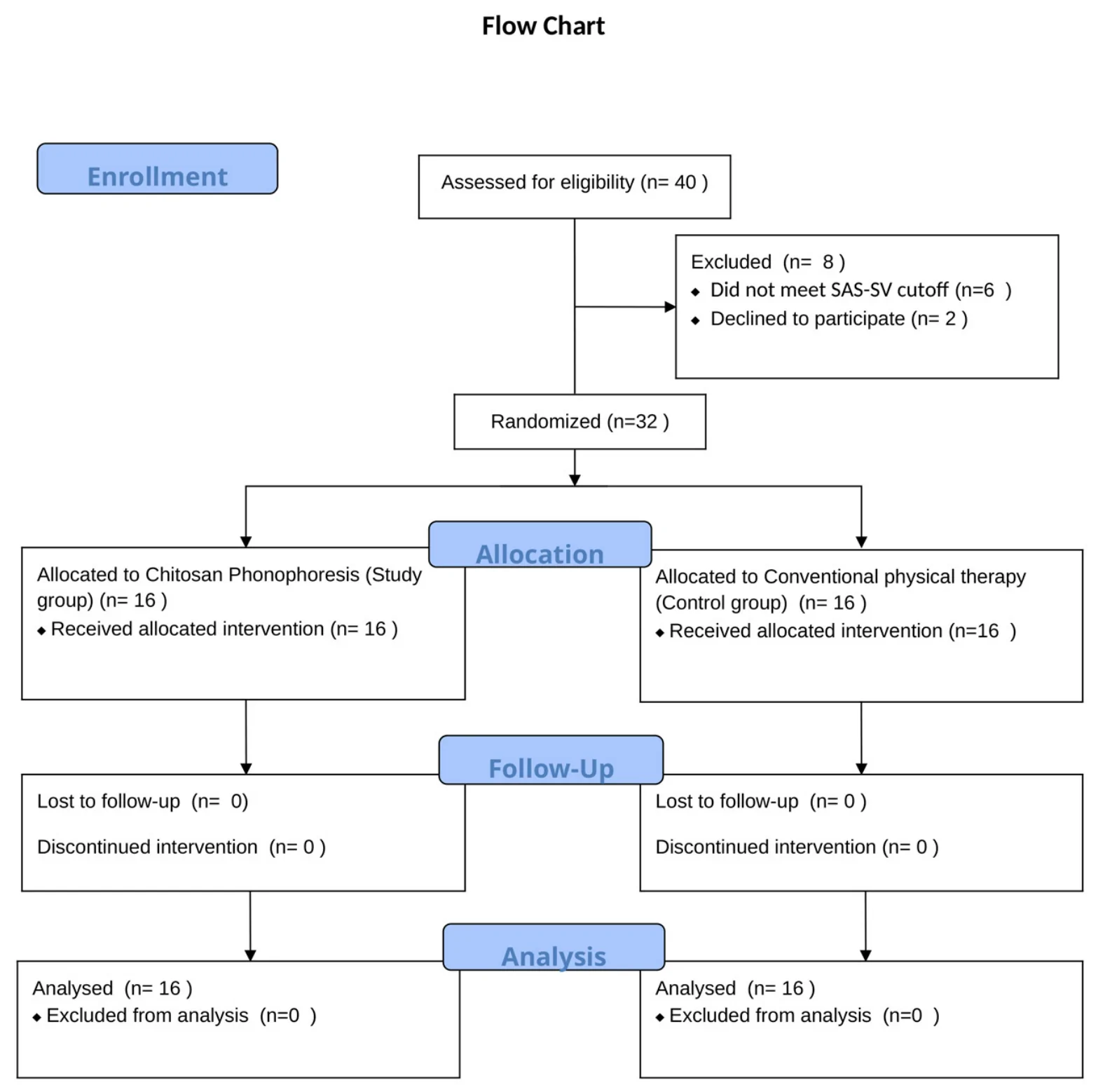
The Flowchart of the whole study.

No participants withdrew after randomization, and all enrolled participants completed the intervention protocol and post-treatment assessment, as shown in Figure 1.

### 2.3. Ethical Consideration

The study was approved by the Ethical Committee of the Faculty of Physical Therapy, October 6 University, Egypt, on 5 June 2024 (Approval No. O6U.PT.REC/024/002003). The trial was subsequently registered at ClinicalTrials.gov on 1 July 2024 (Identifier: NCT06491992), before enrollment of the first participant. The registered protocol specified a BMI range of 25–29 kg/m^2^; however, this criterion was not applied during participant recruitment and was reported only as a baseline anthropometric characteristic. Participant enrollment was conducted from 15 July 2024 to 15 August 2024, and all participants completed the intervention and post-treatment assessment. The ethics approval covered the inclusion of participants younger than 18 years of age. Written informed consent was obtained from the parents or legal guardians of participants younger than 18 years, and written assent was obtained from the participants before enrollment. Participants aged 18 years or older provided their own written informed consent.

### 2.4. Participants

Thirty-two participants with cervical pain were recruited from the outpatient clinic of the Faculty of Physical Therapy, October 6 University Hospital. Smartphone addiction was assessed before enrollment using the Arabic version of the Smartphone Addiction Scale–Short Version (SAS-SV) [19]. The SAS-SV is a 10-item self-report instrument, with each item rated on a 6-point Likert scale ranging from 1 (“strongly disagree”) to 6 (“strongly agree”). The Arabic version has demonstrated good internal consistency and factorial validity, with a Cronbach’s alpha of 0.87. Participants were considered eligible for inclusion if their SAS-SV score met the established gender-specific cutoff of ≥31 for males and ≥33 for females [20]. The inclusion criteria were as follows: participants were aged 16–19 years and included both sexes; had used a smartphone for at least two hours daily for more than one year; had a moderate to severe neck disability score of 15–34 points on the Neck Disability Index (NDI) [21]; and had a pain intensity greater than five on the visual analog pain scale (VAS-P) [22]. BMI was recorded as a baseline anthropometric characteristic but was not used as an eligibility criterion.

Participants were excluded if they had experienced a head or neck injury or undergone head or neck surgery within the preceding year; had congenital abnormalities or symptomatic deformities of the cervical or lumbar spine; had untreated visual or auditory impairments; had headaches, vertigo, or dizziness that could interfere with the assessments; had systemic diseases; had alcohol dependence; or had used addictive drugs or sedatives within 48 h before testing.

### 2.5. Sample Size

G*POWER statistical software (version 3.1.9.2) was used to determine the sample size for this study comparing two groups. The calculation was based on cervical range of motion data reported by Ay et al. [23], in patients with cervical myofascial pain syndrome. An effect size of 1.03, an alpha level of 0.05, a statistical power of 80%, and an allocation ratio of 1:1 were used, resulting in an estimated sample size of 32 participants, with 16 participants in each group. Although cervical range of motion was prespecified as a secondary outcome in the present study, it was used as the basis for the sample-size calculation because the effect-size estimate was derived from the available literature. The effect size was derived from a study involving a different intervention and clinical population and was used as an estimate for sample-size planning.

### 2.6. Outcome Measures

Assessments were conducted before and after the 4-week intervention period. Neck-related disability, assessed using the Neck Disability Index (NDI), and cervical pain intensity, assessed using the Visual Analog Scale for Pain (VAS-P), were considered the primary outcomes. The secondary outcome was cervical range of motion (CROM).

#### 2.6.1. Neck Disability Index (NDI)

The NDI was used to evaluate neck-related disability. The NDI is a well-established instrument with demonstrated validity and test–retest reliability [24]. It consists of 10 sections: pain intensity, personal care, lifting, reading, headaches, concentration, work, driving, sleeping, and recreation. Each section is scored from 0 to 5, resulting in a maximum total score of 50 points. A total score of 0–4 indicates no disability; 5–14 indicates mild disability; 15–24 indicates moderate disability; 25–34 indicates severe disability; and 35–50 indicates complete disability [21].

#### 2.6.2. Cervical Pain

The VAS-P was used to assess pain intensity in the cervical region. The VAS is a horizontal line 100-mm in length with “no pain” at one end and “worst imaginable pain” at the other. Participants were asked to mark a point on the line indicating how much pain they felt at the time of assessment [22]. Previous literature has reported good reliability and validity of the VAS for assessing pain intensity [25].

#### 2.6.3. Cervical Range of Motion (CROM)

Active cervical range of motion (ROM) was assessed using the CROM device (Performance Attainment Associates, Lindstrom, MN, USA) [26].The CROM device is a reliable and valid tool for assessing active cervical ROM in individuals with and without neck pain. Previous research has demonstrated excellent test–retest reliability for the CROM device, with intraclass correlation coefficients ranging from 0.89 to 0.98 across the six cervical movements [27]. Furthermore, good intra-rater reliability has been reported in people with neck pain (ICC 0.88–0.96) [28].

Participants were evaluated in a seated position with the CROM device placed on the head. Active cervical flexion, extension, right and left bending, and right and left rotation were measured in degrees. Participants were asked to actively move their heads through the available range of each movement without using the trunk. Measurements were obtained for each movement and subsequently analysed statistically.

All outcome assessments were performed by a licensed physiotherapist with more than 10 years of clinical experience. The outcome assessor was not involved in participant allocation and was blinded to group assignment throughout the assessment process. A standardized evaluation protocol was provided to ensure consistent assessment procedures across participants.

### 2.7. Intervention

Participants were randomly allocated to two groups. Both groups received the same conventional physical therapy program three times per week for four consecutive weeks at the outpatient physical therapy clinic of the Faculty of Physical Therapy, October 6 University Hospital, under the supervision of a specialized physiotherapist.

The control group received a 10-min ultrasound application using conventional ultrasound coupling gel. Ultrasound was applied to the cervical paravertebral region and upper fibers of the trapezius at a frequency of 1 MHz, an intensity of 1.5 W/cm^2^, and in continuous mode in addition to the conventional physical therapy program for 30 min, with each session lasting for 40 min.

The chitosan phonophoresis group received 10 min of chitosan phonophoresis along with the same conventional physical therapy program. Chitosan gel was applied to the cervical paravertebral region and upper fibers of the trapezius, and ultrasound was delivered at the same frequency, intensity, mode, and duration used in the control group (1 MHz, 1.5 W/cm^2^, continuous mode). The total treatment duration for the chitosan phonophoresis group was approximately 40 min per session.

#### 2.7.1. Chitosan Phonophoresis

For the Chitosan phonophoresis group, chitosan was prepared from Pink shrimp (Meta Penaeus monoceros) shells obtained from local markets were used to extract chitin, following a previously described method [29]. The shrimp shells were carefully cleaned with detergent and water as a pretreatment, and any excess water was then allowed to dry in the sun. Three consecutive processes (demineralization, deproteinization, and deacetylation) were used to transform the dried material into chitosan [30]. The extracted polymer was characterized as having a medium viscosity-average molecular weight *Mw* = 103,200 g/mol with a high degree of deacetylation (97%). The viscosity-average molecular weight molecular weight was experimentally determined via intrinsic viscosity measurements using the Mark–Houwink equation. The degree of deacetylation was determined using Fourier-Transform Infrared (FTIR) spectroscopy (JASCO FTIR 1600 spectrophotometer, JASCO Corporation, Tokyo, Japan) using the spectroscopic method reported by Brugnerotto et al. [31]. To formulate the chitosan gel, 1.75 g of the characterized chitosan was dissolved in 49.8 mL of a 2% (*v*/*v*) acetic acid solution prepared with sterile deionized water, followed by the addition of 0.2 mL of glycerol. The mixture was stirred at 1000 rpm at room temperature for 5 min until a uniform, transparent polymeric gel was formed [32]. To support hygienic preparation and skin compatibility during clinical application, all glassware and equipment were autoclaved, and formulations were prepared extemporaneously under clean, controlled aseptic conditions. The final gel had a pH of approximately 5.0–5.5. Bulk rheological characterization, quantitative microbial limit testing, and long-term stability assessment were not performed in this study.

The cervical paravertebral region and the upper fibers of the trapezius underwent chitosan phonophoresis utilizing an ultrasonic device while the chitosan gel was present. Ultrasound was applied at an intensity of 1.5 W/cm^2^ at a frequency of 1 MHz, in continuous mode to transport the chitosan gel [33], and the therapeutic parameters included a 10-min session length.

Adverse Event Monitoring:

Adverse events and skin safety were monitored throughout the intervention period. During and immediately after each treatment session, participants were assessed for treatment-related symptoms, including skin irritation or redness, burning or thermal discomfort, increased cervical pain, pain flare-ups, or other unexpected adverse effects. Participants were also instructed to report any symptoms or discomfort occurring between treatment sessions.

#### 2.7.2. Conventional Physical Therapy Program

The conventional physical therapy program was designed as a standardized multimodal intervention addressing the main impairments associated with neck pain and disability, including pain, cervical and upper-quarter muscle tightness, restricted flexibility, and impaired cervical postural and motor control. The selection of therapeutic exercises and manual therapy was consistent with clinical practice guidelines and systematic review evidence supporting multimodal physical therapy approaches for the management of neck pain and related disability [9,11].

Each conventional physical therapy session lasted approximately 30 min. The protocol included the following: manual effleurage and kneading massage for the cervical muscles to address cervical soft-tissue discomfort and muscle tightness [34]. The patient was then placed in a supine posture with the head at one end of the table, and the therapist performed stretching exercises for the pectoralis, scalene, and upper trapezius muscles to address soft-tissue flexibility and mobility limitations. Each stretch lasted 30 s, followed by five sets of 30-s relaxation [35]. Cervical manual traction was subsequently performed, with the patient’s head supported by the therapist’s hands near the base of the skull. Traction was applied for 10 s, followed by 5 s of rest, and repeated 10 times [36,37]. The session concluded with postural correction exercises, including cervical chin-in and chin-tuck exercises. These exercises were included to promote cervical motor control and postural awareness. The chin-tuck position was maintained for 5 s, followed by a 5-s return to the starting position [38].

### 2.8. Statistical Analysis

Data were summarized as mean ± standard deviation. Baseline demographic and clinical characteristics were compared between groups using the unpaired *t*-test for continuous variables and the Pearson chi-square test for categorical variables. Normality of data distribution was assessed using the Shapiro–Wilk test.

A multivariate analysis of variance (MANOVA) was performed to assess differences in the outcome measures within and between the study groups before and after the intervention. The NDI and VAS-P were prespecified as co-primary outcomes, whereas CROM was considered as a secondary outcome. A two-sided significance level of 0.05 was used for each co-primary outcome, and no formal multiplicity adjustment was applied. Therefore, the potential inflation of the family-wise Type I error rate associated with testing two co-primary outcomes should be considered when interpreting these findings, particularly for the secondary outcomes, which were considered exploratory. Between-group comparisons at baseline and post-treatment were performed using univariate analyses of variance (ANOVAs). Mean differences were reported with their corresponding 95% confidence intervals (CIs). Within-group pre- to post-treatment changes were also examined. Statistical analysis was performed using SPSS version 20 for Windows (SPSS Inc., Chicago, IL, USA). All tests were two-sided and *p*-value < 0.05 was considered statistically significant.

## 3. Results

### 3.1. Demographic & Clinical Characteristics Data

Table 1 presents the baseline characteristics of the participants. The study included 32 participants, of whom 8 were younger than 18 years of age. There were no statistically significant differences between the Chitosan phonophoresis and control groups in age, body weight, height, BMI-for-age percentiles [39], Smartphone Addiction Scale- short version (SAS-SV), or gender distribution (*p* > 0.05), indicating that the two groups were comparable at baseline.

### 3.2. Normality Test

Before the main analysis, the assumptions of normality, homogeneity of variance, and absence of extreme outliers were examined. The Shapiro–Wilk test showed that all measured variables were normally distributed.

### 3.3. MANOVA Results

A two-way mixed-design MANOVA was conducted to examine the effects of group, time, and the Group × Time interaction across the combined outcome measures. The multivariate main effect of group was statistically significant (Wilks’ Λ = 0.408, F(8, 23) = 4.17, *p* = 0.003, partial η^2^ = 0.592). The multivariate main effect of time was also statistically significant (Wilks’ Λ = 0.008, F(8, 23) = 375.82, *p* < 0.001, partial η^2^ = 0.992). In addition, a statistically significant Group × Time interaction was observed (Wilks’ Λ = 0.144, F(8, 23) = 17.13, *p* < 0.001, partial η^2^ = 0.856), indicating that the overall pattern of change across the combined outcome measures differed between groups.

Follow-up univariate tests of the Group × Time interaction revealed statistically significant interactions for both co-primary outcomes and all six secondary cervical range of motion (CROM) measures. For pain intensity (VAS-P), the interaction was significant, F(1, 30) = 15.38, *p* < 0.001, partial η^2^ = 0.339, as was the interaction for neck disability (NDI), F(1, 30) = 58.28, *p* < 0.001, partial η^2^ = 0.660. Significant Group × Time interactions were also observed for cervical flexion, F(1, 30) = 21.00, *p* < 0.001, partial η^2^ = 0.412; extension, F(1, 30) = 11.78, *p* = 0.002, partial η^2^ = 0.282; right bending, F(1, 30) = 12.83, *p* = 0.001, partial η^2^ = 0.300; left bending, F(1, 30) = 8.63, *p* = 0.006, partial η^2^ = 0.223; right rotation, F(1, 30) = 29.03, *p* < 0.001, partial η^2^ = 0.492; and left rotation, F(1, 30) = 19.80, *p* < 0.001, partial η^2^ = 0.398. These findings indicate that pre- to post-treatment changes differed significantly between the two groups across all outcome measures. The direction of these differences was consistent with greater reductions in pain and disability and greater improvements in cervical range of motion in the chitosan phonophoresis group, as detailed in Section 3.4 and Section 3.5.

### 3.4. Effect of Treatment on Cervical Pain and NDI

There were no significant between-group differences in Visual Analogue Scale for Pain (VAS-P) or Neck Disability Index (NDI) scores before treatment. Univariate ANOVAs before treatment revealed that no significant difference for VAS-P variable, F(1, 30) = 0.11, *p* = 0.741, η^2^ = 0.004, for NDI variable, F(1, 30) = 0.012, *p* = 0.912, partial η^2^ = 0.001.

After treatment, the Chitosan phonophoresis group showed significantly lower pain intensity and disability scores than the control group, univariate ANOVAs post treatment revealed that significant difference for VAS-P variable, F(1, 30) = 12, *p* = 0.002, partial η^2^ = 0.286, for NDI variable, F(1, 30) = 11.39, *p* = 0.002, partial η^2^ = 0.275. Within-group analysis also demonstrated significant improvement in both groups after treatment. Both the Chitosan phonophoresis group and the control group showed significant reductions in VAS-P and NDI scores compared with their pre-treatment values (*p* = 0.001) as shown in Table 2.

### 3.5. Effect of Treatment on Cervical Range of Motion

As presented in Table 3, no statistically significant between-group differences were observed at baseline in cervical flexion, extension, right or left bending, or right or left rotation. The baseline between-group comparisons were not significant for flexion, F(1, 30) = 0.665, *p* = 0.421, partial η^2^ = 0.022; extension, F(1, 30) = 0.253, *p* = 0.619, partial η^2^ = 0.008; right bending, F(1, 30) = 0.000, *p* = 1.000, partial η^2^ = 0.000; left bending, F(1, 30) = 0.293, *p* = 0.592, partial η^2^ = 0.010; right rotation, F(1, 30) = 0.000, *p* = 1.000, partial η^2^ = 0.000; or left rotation, F(1, 30) = 0.000, *p* = 1.000, partial η^2^ = 0.000.

At post-treatment, the chitosan phonophoresis group had significantly greater cervical range of motion than the control group in all six directions. The between-group differences were statistically significant for flexion, F(1, 30) = 14.20, *p* < 0.001, partial η^2^ = 0.321; extension, F(1, 30) = 20.40, *p* < 0.001, partial η^2^ = 0.405; right bending, F(1, 30) = 14.58, *p* < 0.001, partial η^2^ = 0.327; left bending, F(1, 30) = 13.68, *p* < 0.001, partial η^2^ = 0.313; right rotation, F(1, 30) = 25.00, *p* < 0.001, partial η^2^ = 0.455; and left rotation, F(1, 30) = 17.44, *p* < 0.001, partial η^2^ = 0.368. Within-group comparisons showed significant increases in all six CROM directions in both groups after treatment.

Adverse Events:

No treatment-related adverse events were observed or reported in either the chitosan phonophoresis or control group during the 4-week intervention period. No participants required treatment modification, additional treatment, or withdrawal because of an adverse event.

## 4. Discussion

This randomized controlled trial was designed to assess the effects of chitosan phonophoresis combined with conventional physical therapy in smartphone-addicted participants with cervical neck pain. The results indicated that the chitosan phonophoresis demonstrated greater improvement in cervical range of motion (CROM) and significantly greater reductions in cervical pain and Neck Disability Index (NDI) scores compared with the control group which received conventional physical therapy combined with ultrasound using conventional ultrasound gel. These findings suggest that chitosan phonophoresis may provide additional benefits for reducing pain and disability and improving cervical mobility in this population. The observed improvements might be related, in part, to the potential effects of phonophoresis in facilitating the transdermal delivery of topical agents as well as the biological properties of chitosan, which has been investigated for its potential anti-inflammatory, neuroprotective, and tissue-repair effects [40,41].

The analgesic effects observed with chitosan phonophoresis may hypothetically be related to both the phonophoretic delivery of the agent and the biological properties of chitosan. Chitosan possesses several biomedical properties, including anti-inflammatory activity, which may contribute to its potential therapeutic effects [41] In addition, its physicochemical properties and ability to serve as a carrier for therapeutic compounds have supported its application in drug-delivery systems [42]. Recent evidence suggests that chitosan-based phonophoresis may be useful in reducing pain and improving functional outcomes. ElGendy et al. [17] found that nerve conduction velocity, pain intensity and functional outcomes were significantly improved in patients with mild-to-moderate cubital tunnel syndrome who were treated with chitosan phonophoresis relative to those who were treated with conventional therapy. These findings suggest that the combination of chitosan and phonophoresis may enhance the therapeutic effects of chitosan by facilitating its delivery to target tissues. These potential effects may have contributed to the greater reduction in pain intensity and neck disability and the greater improvement in cervical range of motion observed in the chitosan phonophoresis group in the present study. Therefore, the anti-inflammatory and analgesic properties of chitosan, together with its delivery through phonophoresis, may contribute to the beneficial clinical effects observed in patients with postural neck pain. However, the precise mechanisms underlying these effects remain to be fully established.

The improvement in cervical ROM observed in the chitosan phonophoresis group may be partly related to the reduction in pain and neck-related disability. A reduction in pain may enable people to move through a wider available range of movement, whereas pain can restrict voluntary movement and contribute to protective movement patterns and muscle guarding. Previous studies have shown that in individuals with neck pain, there is a reduced active cervical range of motion when compared with asymptomatic individuals [43], and a relationship between cervical range of motion and pain and disability has previously been reported [44]. Therefore, the greater reduction in pain and neck-related disability observed following the addition of chitosan phonophoresis may have facilitated greater cervical mobility. However, this interpretation should be considered cautiously, as the underlying mechanisms were not directly investigated in the present study.

From a clinical perspective, the between-group differences should be interpreted in addition to their statistical significance. The observed between-group difference in pain intensity was approximately 1.0 point on the 10-point VAS, equivalent to 10 mm on a 100-mm scale. Although this difference favored chitosan phonophoresis, it was below the 1.5-point threshold reported by Kovacs et al. [45] for clinically important changes in pain in patients with nonspecific neck pain. Therefore, the clinical relevance of the between-group difference in pain intensity should be interpreted cautiously, despite its statistical significance. Likewise, the between-group difference in NDI was approximately 3.1 points on the 50-point scale. MacDermid et al. [21] reported considerable variability in clinically important differences for the NDI, with approximately 5 points commonly used as a clinically meaningful threshold, although estimates vary according to the population, baseline severity, and methodology. Therefore, although there are statistically significant differences between the groups on pain and disability, the between-group differences in these measures are not necessarily large enough to surpass thresholds for clinically important change. Based on these findings, the possible therapeutic role of chitosan phonophoresis is suggested, and further studies are required to establish whether benefits found in these studies could be extrapolated to clinically meaningful improvements.

The present findings should be considered in the context of smartphone-related neck problems. Previous research has reported an association between prolonged smartphone use and increased neck discomfort [46,47]. Sustained cervical flexion and postural compensations during handheld-device use may increase mechanical loading of the cervical region and contribute to neck pain, functional limitations, and reduced cervical mobility [48,49]. This provides relevant context for the cervical pain, disability, and range-of-motion impairments observed in the participants of the present study.

The improvements observed in both groups may, in part, be attributed to the conventional physical therapy program, which served as the common background intervention in this study. This program included stretching, manual cervical traction, massage, and postural correction exercises, which have previously been associated with improvements in neck pain, cervical mobility, and functional outcomes. Tunwattanapong et al. [50] found that a 4-week program that involved neck and shoulder stretching and postural correction was effective in reduced neck and shoulder pain and improved neck function and quality of life. Similarly, Anderson et al. [51] reported improvements in cervical range of motion and reductions in subjective neck pain following a six-week cervical stretching and strengthening program. However, their study used a single-arm design, so the findings should be interpreted cautiously because changes could not be compared with those in a control group.

One non-invasive method of treating back and neck discomfort is manual traction, which has also been reported to improve pain, cervical mobility, and disability in individuals with neck disorders [36]. Bhutto et al. [37] confirmed that manual cervical traction procedures alleviate neck pain, range of motion, and degree of disability in individuals with cervical radiculopathy and postural correction exercises, such as chin-in exercises, can relieve neck pain in smartphone users [52]. Both groups demonstrated improvements following treatment. However, because all participants received conventional physical therapy, this study cannot establish the independent effectiveness of that program. The between-group differences indicate the relative effects of the two treatment protocols when added to the same conventional physical therapy program. Nevertheless, because the coupling media differed between groups, these differences cannot be attributed specifically to chitosan.

Regarding treatment safety, no treatment-related adverse events were observed or reported in either group during the 4-week intervention period. This finding suggests that both treatment protocols were well tolerated under the conditions of the present study. However, given the small sample size and short intervention period, these findings should not be interpreted as definitive evidence of safety, and larger studies with longer follow-up are needed to further evaluate the safety and tolerability of chitosan phonophoresis.

Several limitations should be acknowledged. The final sample of 32 participants was relatively modest, and the sample size calculation was based on an effect size for cervical range of motion from an intervention and clinical population other than the one used in this study. Therefore, the estimated effect may not fully represent the effect of chitosan phonophoresis in individuals with postural neck pain, and the findings should be confirmed in larger studies. The intervention period was short, and no long-term follow-ups were performed; therefore, it is not possible to determine whether the observed improvements were maintained over time. No vehicle or sham-gel control was used. Although both groups received the same conventional physical therapy program and an equivalent 10-min ultrasound exposure using identical parameters, the coupling medium differed between groups (conventional ultrasound gel versus chitosan gel); however, these differences in coupling mediums between the two groups cannot be completely ruled out. Although the CROM assessor was blinded to group allocation, participants and the treating physiotherapist could not be blinded because the coupling media were distinguishable. Consequently, the self-reported VAS-P and NDI outcomes may have been influenced by participants’ awareness of their treatment allocation, introducing a potential risk of reporting or expectation bias. Furthermore, the study was performed in a single clinical setting and included a relatively young sample aged 16–19 years with variability in BMI, which may limit the generalizability of the findings to other age groups and populations. In addition, bulk rheological properties, quantitative microbial limit testing, and long-term stability of the extemporaneously prepared chitosan gel were not evaluated. Therefore, the formulation characteristics and stability under prolonged storage were not established. There is a need for larger multicenter trials with longer follow-ups and appropriate vehicle controls with prespecified outcomes.

## 5. Conclusions

Adding chitosan phonophoresis to conventional physical therapy was associated with greater improvements in cervical range of motion and statistically greater reductions in cervical pain and disability than conventional physical therapy combined with ultrasound using conventional gel among smartphone-addicted users aged 16–19 years. However, the between-group differences in pain and disability were below commonly used thresholds for clinical importance, and interpretation of these differences is further limited by the lack of participant blinding. Because no vehicle-gel control was used, the additional effects cannot be attributed specifically to chitosan rather than to differences between the coupling media or other treatment-related factors.

## Figures and Tables

**Table 1:** Baseline demographic and clinical characteristics of the study and control groups.

Variables	Chitosan Phonophoresis Group (*n* = 16)	Control Group (*n* = 16)	t-Value	*p*-Value
	Mean ± SD	Mean ± SD		
Age (years)	17.75 ± 0.46	17.88 ± 0.64	−0.45	0.662
Height (cm)	166 ± 5.13	167.25 ± 3.28	−0.88	0.387
Weight (kg)	70 ± 6.09	70.25 ± 6.85	−0.11	0.914
BMI-for-age percentiles	81.85 ± 9.43	77.54 ± 15.1	0.97	0.341
SAS-SV	41.88 ± 4.49	41.75 ± 5.5	0.05	0.961
Gender (%)				
Male	7 (43.75%)	8 (50%)	χ^2^ = 0.12	0.723
Female	9 (56.25%)	8 (50%)		

Values are presented as mean ± standard deviation (SD) unless otherwise indicated. BMI = Body Mass Index; SAS-SV = Smartphone Addiction Scale-short version; χ^2^ = Pearson chi-square test; *p* < 0.05 indicates statistical significance.

**Table 2:** Pre- and post-treatment comparison of cervical pain and neck disability in both groups.

Variables	Chitosan Phonophoresis Group (*n* = 16)	Control Group (*n* = 16)	MD (95% CI)	*p*-Value	Partial η^2^
	Mean ± SD	Mean ± SD			
VAS-P					
Pre	7 ± 0.92	6.88 ± 1.25	0.12 (−0.64, 0.89)	0.741	0.004
Post	2.5 ± 0.93	3.5 ± 0.76	−1 (−1.59, −0.41)	0.002 *	0.286
*p*-value	0.001 *	0.001 *			
NDI					
Pre	23.63 ± 3.16	23.5 ± 3.42	0.12 (−2.17, 2.42)	0.912	0.001
Post	15 ± 2.45	18.13 ± 2.95	−3.13 (−5, −1.23)	0.002 *	0.275
*p*-value	0.001 *	0.001 *			

Values are presented as mean ± standard deviation (SD); VAS-P, Visual analogue scale for Pain; NDI, Neck Disability Index; CI = confidence interval; η^2^, partial eta square; * *p* < 0.05.

**Table 3:** Pre- and post-treatment comparison of cervical range of motion in both groups.

Variables	Chitosan Phonophoresis Group (*n* = 16)	Control Group (*n* = 16)	MD (95% CI)	*p*-Value	Partial η^2^
	Mean ± SD	Mean ± SD			
Flexion					
Pre	35.63 ± 4.03	34.38 ± 4.62	1.25 (−1.88, 4.38)	0.421	0.022
Post	44.38 ± 4.03	38.75 ± 4.4	5.63 (2.58, 8.67)	<0.001 *	0.321
*p*-value	0.001 *	0.001 *			
Extension					
Pre	46.25 ± 3.42	45.63 ± 3.61	0.62 (−1.91, 3.16)	0.619	0.008
Post	58.13 ± 2.5	51.88 ± 4.94	6.25 (1.90, 10.59)	<0.001 *	0.405
*p*-value	0.001 *	0.006 *			
Right bending					
Pre	33.13 ± 3.59	33.13 ± 4.69	0 (−3.01, 3.01)	1.000	0.000
Post	44.38 ± 1.71	40.63 ± 3.54	3.75 (1.74, 5.76)	<0.001 *	0.327
*p*-value	0.001 *	0.001 *			
Left bending					
Pre	35.63 ± 3.1	35 ± 3.43	0.63 (−1.73, 2.98)	0.592	0.010
Post	44.38 ± 1.71	40 ± 4.41	4.38 (1.95, 6.79)	<0.001 *	0.313
*p*-value	0.001 *	0.005 *			
**Right rotation**					
Pre	51.25 ± 2.41	51.25 ± 3.17	0 (−2.03, 2.03)	1.000	0.000
Post	63.75 ± 3.42	57.5 ± 3.65	6.25 (3.70, 8.80)	<0.001 *	0.455
*p*-value	0.001 *	0.001 *			
**Left rotation**					
Pre	52.5 ± 1.55	52.5 ± 2	0 (−1.29,1.29)	1.000	0.000
Post	64.38 ± 4.03	58.13 ± 4.43	6.25 (3.19, 9.30)	<0.001 *	0.368
*p*-value	0.001 *	0.002 *			

Values are presented as mean ± standard deviation (SD); CI = confidence interval; Partial η^2^ = partial eta square; * *p* < 0.05.

## Data Availability

The data presented in this study are available on request from the corresponding author because they contain information that could compromise participant privacy and are subject to ethical restrictions.

## Supplementary Materials

The following are available online at https://www.mdpi.com/article/10.3390/healthcare14213626/s1, Table S1: CONSORT 2025 checklist of information to include when reporting a randomised trial*.
